# Supplementary material for: Photovoltaic Device Application of a Hydroquinone-Modified Conductive Polymer and Dual-Functional Molecular Si Surface Passivation Technology
Source: Polymers (Basel). 2022 Jan 25;14(3):478. doi: 10.3390/polym14030478 (PMC8839862; doi:10.3390/polym14030478)
Supplement: Supplementary file 1 [file polymers-14-00478-s001.zip › polymers-1568069-supplementary.pdf]

polymers

MDPI

Supplementary Materials

Photovoltaic Device Application of a Hydroquinone-Modified Conductive Polymer and Dual-Functional Molecular Si Surface Passivation Technology

Na Yeon Park [1], Gwan Seung Jeong [1], Young-Jin Yu [1], Yoon-Chae Jung [1], Jin Hee Lee [2], Jung Hwa Seo [2] and Jea-Young Choi [3,*]

1 Department of Metallurgical Engineering, Dong-A University, 37 Nakdong-Daero, 550 Beon-gil, Saha-gu, Busan 604-714, Korea; nayeon2385@gmail.com (N.Y.P.); wjdrhkstmd12@gmail.com (G.S.J.); tq6284@naver.com (Y.-J.Y.); dbsco0306@naver.com (Y.-C.J.)
2 Department of Chemical Engineering, Dong-A University, 37 Nakdong-Daero, 550 Beon-gil, Saha-gu, Busan 604-714, Korea; dljh82@gmail.com (J.H.L.); seojh@dau.ac.kr (J.H.S.)
3 Department of Materials Sciences & Engineering, Dong-A University, 37 Nakdong-Daero, 550 Beon-gil, Saha-gu, Busan 604-714, Korea
* Correspondence: cjy4395@dau.ac.kr

$$J = J_0(\exp\left(\frac{eV}{nkT}\right) - 1) \quad (1)$$

$$J_0 = A^{*}AT^2 \exp\left(-\frac{V_{bi}}{kT}\right) \quad (2)$$

We measured the dark J-V curve to analyze the characteristics of the HSCs interface additionally. Diode equations such as Equations (1) and (2) can express the dark JV characteristic in log scale and extract the values of the desired characteristics. A is the contact area, A* is the effective Richardson constant (120 $Acm^{-2}K^{-2}$ for *n*-type silicon), T is 298 K (25 °C), k is Boltzmann constant, n is ideality factor, J_0 is the reverse saturation current density, V_{bi} is the barrier height in Schottky diodes, i.e., built-in-potential, and q is the elementary charge.

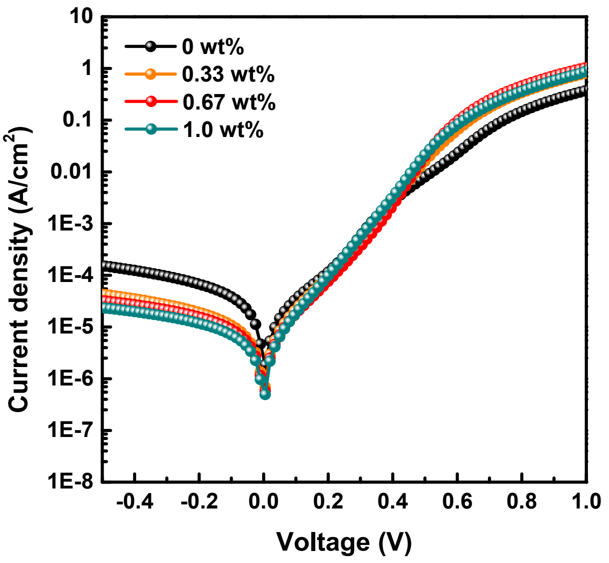

Figure S1. ln J-V curves measured in the dark condition for fabricated *n*-Si/HQ-PEDOT:PSS HSCs with various HQ addition amounts.

Table S1. Summary of J_0, *n*, and V_{bi} fitted parameters from the measured dark ln J-V curves of various amounts of Si/HQ-PEDOT:PSS HSCs.

Amount of HQ addition	J_0 (A/cm^2)	n	V_{bi} (V)
0 wt%	1.58×10^{-5}	3.39	0.627
0.33 wt%	3.69×10^{-5}	2.63	0.665
0.67 wt%	4.39×10^{-6}	2.70	0.661
1.0 wt%	3.54×10^{-6}	2.32	0.666

Table S2. Summary of J_0, n and V_{bi} fitted parameters from the measured dark ln J-V curves of the Si PEDOT:PSS(DMSO, HQ) HSCs.

PEDOT:PSS	J_0(A/cm^2)	n	V_{bi} (V)
DMSO	1.54×10^{-5}	2.82	0.623
HQ	4.39×10^{-6}	2.70	0.661

The dark J-V curve was measured to analyze the characteristics of the BQ-HSCs interface additionally. According to the contact angle with τ_{eff}, the leakage current value of BQ-HSC should decrease more than that of SiOx-HSC. As shown in Figure S2, the leakage current in the reverse bias is significantly smaller than SiOx-HSC, indicating a decrease in carrier recombination at the interface. The desired dark J-V characteristics values in the log scale were extracted using Equations (1) and (2)

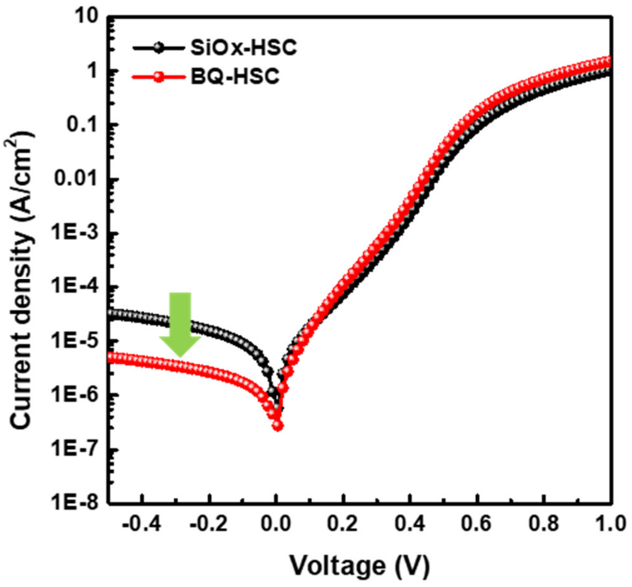

Figure S2. Dark characteristics (a) Dark ln J-V curves, (b) d(V)/d(ln J) vs J plots and (c) H(J) vs J plots of the BQ-HSC and SiOx-HSC.

The n value of HCSs can be extracted from the slope of the tangent line corresponding to the log scale J-V curve in the forward voltage section of 0.1–0.2 V, and the n value of SiOx-HCS and BQ-HSC was calculated using Eq. (S1). BQ-HSC has a lower value than SiOx-HSC, representing a better junction quality because of the improved wettability due

to the BQ passivation layer between HQ-PEDOT:PSS and Si surface. Moreover, $J_{.0}$ is a value determined by carrier recombination in the neutral region on both sides of the junction. As mentioned above, the J_0 value of HQ-HSC is smaller than that of SiOx-HSC due to the reduction in carrier recombination on the Si surface because of the chemical passivation on the Si surface and the field effect passivation by dipole formation. Equation. (2) can extract V_{bi} from the J_0 value. Therefore, the characteristic result of the dark JV becomes the basis for high photovoltaic efficiency due to the efficient carrier transport and collection.

Table S3. Summary of J_0, n, and $V_{bi,}$ fitted parameters from the measured dark ln J-V curves of the HSCs.

Passivation	J_0(A/cm^2)	n	V_{bi} (V)
SiOx-HSC	4.39×10^{-6}	2.70	0.661
BQ-HSC	1.25×10^{-6}	2.03	0.693
